# Combining transnational and intersectional approaches to immigrants' social protection: The case of Andean families' access to health

**DOI:** 10.1186/s40878-018-0073-7

**Published:** 2018-06-04

**Authors:** Jean-Michel Lafleur, Maria Vivas Romero

**Affiliations:** Centre for Ethnic and Migration Studies (CEDEM), Université de Liège, Quartier Agora, Place des Orateurs 3, 4000 Liège, Belgium

**Keywords:** Gender, Transnationalism, Social protection, Health, Peru, Colombia

## Abstract

Immigrants and family members in the home and host societies experience inequalities in access to social protection. Focusing on healthcare, we demonstrate that immigrant families today respond to healthcare needs of family members here and there through four cross-border strategies. We show that immigrants select and articulate these different strategies to assemble transnational health care arrangements. Using an intersectional approach, we argue that heterogeneity markers such as gender, race, class, and levels of transnational engagement determine the choice between different types of arrangements. We support our argument with ethnographic data collected with 48 members of 10 Andean transnational family members during fieldwork in Belgium, Colombia, and Peru.

## Introduction

The nexus between migration, healthcare, and social protection has received significant scholarly attention over the past decades. Some public health scholars, for instance, have looked for a long time at the role of health as a driver for migration and, conversely, at the impact of migration and the condition of being an immigrant on health and access to formal care (Lindert, Schouler-Ocak, Heinz, & Priebe, 2008; Bollini & Siem, 1995). Migration scholars, on the other hand, have had an interest in immigrants as care providers in destination countries and its impact both on family ties across borders (Baldassar & Merla, 2014) and on the availability of care in the Global South (Isaksen, Devi, & Hochschild, 2008). Overall, because these academic conversations have developed separately, scholars have for the most part neglected the fact that the immigrant families organize their own access to health across borders.

However, recent literature on transnational social protection has instead partly helped to bridge the gap between these different bodies of literature. In particular, they found that transnational families access social protection through formal schemes in sending and receiving countries as well as through informal provisions based on social networks located in multiple geographical locations (Sabates-Wheeler & Feldman, 2011; Faist, Bilecen, Barglowski, & Sienkiewicz, 2015). In this article, we first build on the above-mentioned bodies of literature but also on social policy, development and diaspora studies in order to identify four strategies by which immigrants ensure their family’s and their own access to formal healthcare. Adopting a transnational-intersectional lens (Anthias, 2008; Mahler, Mayurakshi, & Vrushali, 2015), we argue —in the second part of the paper— that these avenues are not equally open to all immigrants as their availability is determined by various heterogeneity markers such as gender, race, class, and levels of transnational engagement.

We put to use an intersectional approach first developed by self-described “U.S third-world feminist” (Moraga & Anzaldúa, 1983) to analyze the multiple axes of inequalities beyond gender that create mechanisms of inequality in contemporary societies. Following pioneer work by Crenshaw (1991) and its increasing use in Europe since the 1990’s (Anthias, 2008), intersectionality has undoubtedly been transformed into a popular heuristic device in contemporary social sciences. However, as argued by migration scholars such Mahler et al. (2015), it has not reached its full analytical potential given that most scholars apply it to understand power relations inside one particular nation-state. In spite of the fact that an increasing number of individuals are living lives that span across the borders of nation-states, a certain form of methodological nationalism remains (Wimmer & Glick-Schiller, 2002). In recent years, sociologists like Purkayastha (2010, p. 40) advocated an intersectional approach that could reveal how exclusion and inclusion work in different national contexts. Indeed, because markers of heterogeneity such as gender, class, and ethnicity produce different effects whether they are looked from the standpoint of the immigrant’s sending or receiving country, we need to go beyond what some have called “domestic” intersectional studies (Patil, 2013). As we show with our case study, because the immigrant position in term of class, gender and races produces different effects in the sending and receiving country, immigrant families may actively design their social protection strategies to counterbalance the less advantageous position they have in one space with a more privileged position they have in the other. This understanding of simultaneous experiences of multiple standpoints might help us to understand inequalities in a transnational context characterized by the geographical separation of immigrant family members and their simultaneous social, economic and political involvement in different nation states.

We thus build on Purkayastha’s work (2010) to study transnational healthcare arrangements used by Andean transnational family networks. Such arrangements are made of various strategies by which access to formal healthcare is negotiated by immigrant families across the borders of several nation-states. Beyond the classic “gender, race and class trilogy” adopted in intersectional studies, we argue that a thorough understanding of transnational health care arrangements by immigrant families need to also take in consideration additional heterogeneity markers such as religiosity, generation and their level of transnational engagement. Following the work of Frenoza-Flot and Shinozaki (2017, p. 8), we analyzed generation not as a biological category but as the historical location of an individual within his/her family network, which might change meanings in different national or social contexts.

Andean migrants have traditionally been considered as a homogeneous group of migrants with low access to social protection. Yet, the analysis of our empirical data reveals that these families are heterogeneous units who select specific strategies and assemble them in two types of transnational health care arrangements —sequential and sporadic arrangements— according to different heterogeneity markers. By conducting such analysis we go beyond the work of transnational feminist scholars who have used an intersectional lens in a global set of relations particularly applied to identity formations or to the mapping of how ideas and capital get transferred across borders through different power lines but rarely to study the distribution of social rights (Grewal & Kaplan, 1994).

### A typology of immigrants’ transnational health strategies

How do immigrants organize their own access to healthcare and that of relatives living with them or in the home country? For over two decades, transnational migration scholars have studied the interconnectedness between immigrant well-being and that of relatives from whom they are physically separated. With concepts such as transnational care (Baldassar & Merla, 2014) and care chains (Hochschild, 2000), in particular, they have shown how the mobility of migrants driven by labor shortages in the care sectors of industrialized countries itself triggers new needs for care in the societies of origin (Yeates, 2009). These works expanded the meaning of care to embrace various forms of cross-border material and moral support that circulate across transnational family networks and are governed by family and kinship ties that are not gender-neutral (Baldassar & Merla, 2014). By extending the meaning of care to informal practices, these scholars have however neglected the new avenues to access formal health care that have recently developed. For this reason, while we recognize that formal strategies often intertwine with informal ones, this article focuses on access to formal care. To this end, we use the narrower concept of immigrants’ transnational health strategies, which are the rights, schemes, and practices available to immigrants to provide both themselves and relatives in the host, and home societies with access to medical treatment from qualified health professionals.

Access to formal healthcare and welfare, in general, has been historically gender- biased. In the classic male breadwinner model common to many Western states, women were traditionally in charge of households’ reproductive needs while the family’s access to state-sponsored welfare depended on male workers (Sainsbury, 1999). Next to gender biases, migration regimes also historically created distinctions between categories of immigrants with guaranteed access to health (e.g. mobile EU citizens, refugees…) and others —such as undocumented migrants— with limited rights to welfare (Sainsbury, 2006). Scholars have noted, however, that in Europe in particular these lines are now blurred by the increasing use of health policies as migration control instruments (Gsir, Lafleur, & Stanek, 2015). Bearing these limitations in mind, we identify four transnational health strategies below before discussing, with the case study of Andean transnational families, how immigrants select them to create what we call their own transnational health care arrangements (see Table 1).

**Table 1:** A Typology of immigrant transnational health strategies

1. Worker’s Insurance	2. Mobility
Public or private health insurance covering immigrants in situation of employment with (possible) extension of benefits to relatives residing in the home country	Cross-border movement of immigrant towards homeland or third country to receive care and cross-border movement of immigrant relatives to receive care in immigrant country of residence
3. Individual and Collective Remittances	4. Diasporic Health Policies
Cash transfer from immigrants to non-migrant relatives to purchase access to healthcare in the home country and community-based forms of solidarity by which immigrants and/or relatives in the homeland are provided with means to access healthcare	Ad hoc policy responses by home country authorities to address specific healthcare needs of nationals with limited or no access to healthcare abroad with (possible) extension of benefits to non-migrant relatives residing in the home country

### Workers’ health insurance for immigrants and dependents abroad

Public or private health insurance purchased in countries of residence is a favored avenue for immigrants wishing to address their family’s health needs here and there. While these insurances typically focus on the needs of residents, medical expenditures of relatives in the homeland can sometimes be covered. Social security agreements —whenever they are signed between sending and receiving country authorities— are key instruments in this respect as they allow immigrant workers to extend health insurance benefits in their country of residence to family members abroad (Sabates-Wheeler & Koettl, 2010). Because of the high level of coordination between social security institutions of Member States, EU citizens are among the most privileged immigrants when it comes to extending health care coverage to relatives in the homeland. With Regulations 883/2004 and 987/2009, the state where migrants are employed reimburses the state where the family is residing for costs incurred (Holzman, Koettl, & Chernetsky, 2005). The European model is however exceptional as most immigrants have limited ability to export social security coverage to relatives residing abroad because of their legal status or the absence of such social security agreements (Holzman et al., 2005).

Next to public health insurance, numerous health insurance companies offer costly “expatriate insurances” on the private market that cover socio-economically privileged immigrants residing abroad and their family members located in multiple geographical spaces. In California, experiments have also been conducted to allow US employers to subscribe to cheaper private insurance plans for documented immigrant workers and family members in Mexico. This plan gives migrants healthcare in Mexican border cities at lower cost (Vargas-Bustamante, Laugesen, Caban, & Rosenau, 2012). Overall, the migration status, as well as bureaucratic and financial barriers often hampers immigrants’ ability to extend public or private health coverage to family members abroad. Additionally, such provisions rely on western-based family conceptions limiting themselves to spouse, children and parents even though migrants’ kinship obligation may extend beyond the nuclear family.

### Mobility

Immigrants who have limited coverage of private or public health insurances often engage in mobility strategies to take care of both their healthcare needs as well as those of non-migrant family members. Transnational family scholars have analyzed how family members residing in different countries organize healthcare across borders. As demonstrated by Merla and Baldassar (2011), gendered and generational markers of difference matter in this distribution, which entails for instance, that women or younger siblings may be granted different responsibilities when it comes to the family’s health.

Mobility as a transnational health strategy also materializes differently for migrant and non-migrant family members. First, immigrants in need of healthcare may return temporarily or permanently to their home country or a third country in which they lived previously or where medical care can be accessed for lower prices (Bilecen & Tezcan-Guntekin, 2014). Sending countries such as Poland, Croatia or Morocco who grant preferential access to their public health system to permanent or temporary return migrants facilitates this strategy. Second, immigrants could organize their relatives’ temporary or permanent relocation to their new country of residence where their health insurance policy allows for the coverage of relatives living in the same household. As noted by Godin (2013) mobility of sick relatives can be facilitated through medical visas and family reunification. Indeed, relatives that visit family members in sending countries on tourist visas in destination countries may also receive medical treatment abroad. In this case, however, treatment is usually paid in full to practitioners without any social security intervention. Overall, as most industrialized nations are concerned with the protection of their borders and welfare systems, getting a visa is often a time- consuming and uncertain process. They, thus often represent an unsatisfactory response to situations when recurrent, urgent or serious care is needed.

### Individual and collective remittances

In sending societies, remittances play a key role in the ability of the immigrant family member to access health care. Indeed, in countries where public health systems are weak, incomes generated by migration are often used to purchase medical treatment directly with providers or to purchase a private health insurance (Kabki, 2007). As the interest in the impact of remittances on sending societies has grown tremendously in the past decade, we now have evidence that remittances improve family members access to formal healthcare and facilitate the purchase of treatments (Lopez-Cevallos & Chi, 2012).

Alongside the research on individual remittances, migration and development scholars have also noted that collective remittances can improve relatives’ access to healthcare in the home country. Numerous examples exist of immigrant groups pulling resources together to respond to individual or collective needs in the home country by financing finance health or educational infrastructures in their hometowns (Goldring, 2004; Østergaard-Nielsen, 2009). It is noteworthy that remittance sending is not a gender-neutral practice either. Scholars have noted that sending remittances can transform women in breadwinners from afar, therefore questioning traditional role within families (Pribilsky, 2004). This, however, sometimes occurs at the expense of immigrant women’s own access to healthcare (Soresen, 2004).

Next, to financial remittances, non-material systems of transnational exchanges also exist. Levitt called social remittances the ideas, norms, and behaviors that circulate between sending and receiving societies through migration (Levitt & Lamba-Nieves, 2011). In the case of health, ‘social remittance’ is a useful concept to capture the informal practices that immigrants perform to help their relatives abroad access healthcare. Such practices do not involve financial transactions (or not immediately) but improve relatives’ access to formal health care. This is the case of immigrants who identify and pay practitioners in the homeland from abroad to facilitate relatives’ access to medical treatments (see, e.g., Boulanger, 2014) on Malian migrants in France). Immigrants’ exposure to foreign healthcare systems may increase their expectations about public health systems and enable them to identify good practitioners in the home country and build trust with them. Social remittances, however, do not de facto positively impact relatives’ health as harmful behaviors and practices can also be transmitted across borders (see, e.g., Flórez, Dubowitz, Saito, Borges, & Joshua, 2012 on obesity).

At the cross-road between financial and social remittances, different migrant communities have also taken steps to create their own insurance schemes to cover health needs of relatives abroad. This is, for instance, the case of Solidarco, insurance fund created by Congolese migrants in Belgium in cooperation with a Belgian insurance company and a network of Congolese health practitioners. Doing so, no only do they respond to health care needs abroad, they also limit their own remittance spending (see, e.g. Lafleur & Lizin,  2015).

Overall, as noted by Mazzucato (2011), the act of sending family remittances pertain to wide support networks. In such systems of reciprocal obligations, relatives in the homeland who receive money may also send “reverse remittances” (mainly in the form of services) to help migrants in need. Therefore, financing relatives’ access to medical services is often a form of investment for migrants. As we will discuss in the case of Andean transnational family networks, those who receive remittances today may also be those who facilitate aging immigrants’ access to healthcare tomorrow. In this sense, we postulate that adopting an intersectional approach and looking at the generation, gender, race and transnational engagement of both senders and recipients of remittances is an indicator of long-term strategies to access health care.

### Diasporic health policies

In the more recent literature on diaspora and transnationalism, scholars have noted that immigrants’ countries of origin are increasingly willing to engage with citizens abroad and address some of their needs (Ragazzi, 2014). Among the variety of “diasporic policies” (Smith, 2003), there exist a series of health-related initiatives following sending countries’ governments concern with their expatriates’ access to healthcare. Several Latin American consulates, for instance, actively campaign to improve immigrants’ access to formal healthcare schemes in the US and sometimes even provide access to medical services on their premises (Délano, 2014).

Diasporic health policies —because there are focused on responding to immigrants’ needs here and there— can also comprise of specific health coverage schemes that cover both immigrants and relatives in the homeland. The Philippines and Sri Lanka, for instance, have set up welfare funds that cover disabilities and diseases incurred by immigrants while working abroad and, in certain cases, extend medical coverage to family members of immigrants who stay in the home country (Mackenzie, 2005). Other states like Mexico have taken specific provisions to include immigrants and non-migrant family members in existing social security schemes. With its universal health coverage scheme called Seguro Popular not only can immigrants prepare for their own access to Mexican public health upon return, they can start registering non-migrant relatives from abroad (Vargas-Bustamante et al., 2012).

In spite of the growing interest of sending states for citizens abroad, gender-specific policies remain few with the exception of sending state’s protection against specific health hazard (e.g. exposure of domestic workers to abuse from employers). For the most part, diasporic policies do not take into consideration the specific needs and gender obligations of male and female migrants when it comes to organizing health within transnational families.

### Situating Andean family network’s transnational healthcare arrangements

Early waves of Andean migration during the twentieth century were strongly connected to homeland conflicts but this situation evolved dramatically in the 1990s. By then, Andean migrants arriving mostly in Spain and Italy were no longer political refugees but increasingly women who took on a migratory project to provide for the economic and social welfare of their families. Their move was mostly triggered by continuous political and economic crises in their countries of origin (Martinez-Franzoni, 2008) as well as by growing needs for domestic and care workers in Western countries, and particularly in Southern Europe (Escrivá & Díaz-Gorfinkiel, 2011).

In recent years, these gendered-migratory flows have diversified in terms of destination countries within Europe as restrictive migration policies and the economic crisis have forced Andean transnational families to opt for alternative destinations. In countries like Belgium they also found employment in the care and domestic work sector (Freitas & Godin, 2013; Camargo, 2015)^1^ and their access to formal social protection like in other destination countries is strongly determined by their legal status and work status. For this reason, Andean female migrants who are undocumented or work in the informal care economy are de facto excluded from most public healthcare programs except emergency care. In addition, recent budget cuts in welfare programs, increased stigmatization of immigrants as “welfare abusers”, restrictions in access to family reunification, medical visas, and Belgian nationality have considerably reduced the avenues by which immigrants can bring sick relatives to Belgium (Lafleur & Stanek, 2017).

Looking at our networks’ countries of origin, it is to be noted that Peru and Colombia, are “familialist welfare states” which entails that families in those countries have traditionally had to rely on the unpaid reproductive labor of female family members for informal healthcare, as well on other private options (Martinez-Franzoni, 2008). In addition to recent innovations in terms of universal healthcare policies, Colombia, however recently created the online platform “Colombia Nos Une” (Colombia Unites Us) with the specific purpose of assisting emigrants in their access to social protection abroad (Ministerio de Protección Social-Colombia, 2010; Velazquez, Suarez, & Nepo-Linares, 2016; Colombia-Nos-Une, 2014). Through this platform, Colombian authorities organize health campaigns by which Colombian nationals in Europe, North America, and Latin America can access medical, psychological and dental check-ups (Colombia-Nos-Une, 2016).

Overall, recent changes in sending and receiving states migration and welfare policies clearly affect immigrants’ options when it comes to addressing health challenges. However, as transnational health strategies are being reconfigured, we argue that not all Andean immigrants have equal opportunities to opt for one strategy or another. Using the concept of transnational social protection arrangement, we intend to show that heterogeneity markers determine the selection of health strategies. The concept of transnational social protection arrangement builds on earlier work on “assemblages” that considered that access to social protection through informal support resources embedded in interpersonal relations and social policy regulations reproduce and produce new intersecting inequalities globally (Faist et al., 2015). With the concept of arrangement, we want to stress that these are “fluid processes” embedded in welfare, work and care regimes of various states. These arrangements allow family members to access social protection and change according to the availability of resources at particular moments of the life-course. Arrangements —unlike assemblages described earlier by Amelina, Bilecen, Barglowski, and Faist (2012)— stress the individuals’ agency in adopting certain strategies over others. Transnational social protection arrangements are thus intertwined strategies determined by markers of difference such as age, gender, class, religiosity or transnational engagement by which transnational families negotiate their access to formal healthcare in the home and/or host societies.

## Methods and research design

### Sample profile

Our analysis builds on transnational healthcare arrangements used by 10 Andean transnational family networks. Author B collected this data during her Ph.D. thesis under the close supervision of author A. The participants were initially selected through a snowball technique in various points of entry in Brussels such as churches of different denominations and cultural events. These multiples points of entry ensured appropriate diversity in terms of intersectional markers of difference mentioned above. Female migrants —who tend to be the majority of Peruvians and Colombians in Brussels— were contacted first. This population usually spent extensive periods abroad and, as they are aging, they find themselves worrying equally about their families’ as well as their own access to social protection.

### Data collection strategy and methods

To collect the data, author B adopted “a mutated witness” approach by which she discretely observed and learned from the participants as she built a testimony of their strategies (Haraway, 1997). In other words, during her 20-month long multi-sited ethnographic fieldwork, she let the participants guide her through fieldwork and tell her about their transnational healthcare arrangements. In line with our ethnographic approach, life-stories quickly appeared as an appropriate instrument to collect the data as they allow participants to situate their strategies within their life-course (Bruner, 1987). Building on their life-stories, Brussels-based informants identified actors in their family networks whom they considered key in their negotiations to access social protection (Widmer, Aeby, & Sapin, 2013). Author B accordingly conducted 31 additional life-story interviews with male and female family members in the immigrants’ home communities in Colombia (Bogota and Medellin and Peru (Lima and Chimbote). Similarly, participants also identified other community and civil servant actors both in Brussels and in their country of origin with whom we conducted 17 semi-structured interviews (Hopf, 2004). In addition to life-stories and semi-structured interviews, author B conducted extended stays in the informants’ homes in Brussels as well as with relatives in Colombia and Peru. All the participants’ names cited in this articles have been changed in order to guarantee anonymity.

## Results

During our fieldwork, our participants were invited to discuss their transnational healthcare strategies. Through this process, we were able to co-construct the process that led to the negotiation of specific transnational social protection arrangements. More precisely, we identified two types of social protection arrangements —sequential and sporadic arrangements— that reflect the different opportunities available to immigrants and family members within immigrant groups such as Andean migrant domestic workers (hereafter referred to as MDW) that could appear as relatively homogenous at first sight.

### “Today for you tomorrow for me”: Sequential arrangements

Existing scholarship has shown that, within Andean transnational family networks, the first step towards international mobility is usually the departure of a pioneer female migrant moving internally from rural areas of Peru or Colombia to larger urban centers (Escrivá & Díaz-Gorfinkiel, 2011). In the city, female migrants can typically find employment as domestic workers. Our informants are no exception in this respect as their first internal move as a family was often followed by international migration of a younger member of the family. In those internal and international migratory paths, health plays a key role both as a trigger for mobility but also as an opportunity to access care that is not available in the home communities.

Mobility to access healthcare can occur in different directions depending on the location of a family member in need of such care. First and foremost, immigrants seek to bring over sick relatives to destination countries where the immigrants themselves can organize their access to care. This is the case of one of our informant named Mariana —the mother of a Peruvian migrant domestic worker residing in Belgium— who has move repeated both internally and internationally to receive healthcare. During her interview, she recalls how mobility for health reasons is repeated generation after generation within the family:

(…) My mother migrated from the mountains [to the city]. She had no healthcare, no insurance, no nothing (...). [Later,] I helped my own daughters to migrate (...). Now, they take me to Belgium to be taken care of… That’s how they found out I have an awful bacteria that could kill me (…). My daughter also took [my grand-daughter] Clarita to Belgium. She had cancer and got cured. (Mariana, 70, mother of MDW2, residing in Lima).

Using mobility to address immediate healthcare needs of relatives is however strongly determined by the immigrant’s employment and migratory status. Undocumented migrants but also documented MDWs working under specific regulations for diplomatic staff often see their mobility options limited: family members living in the homeland cannot come to them to receive healthcare and they themselves cannot return temporarily to the country of origin to assist family members. The story of Solimar, a MDW who could not bring her sick mother over to receive care illustrates this difficulty:

“God knows for how long she had been suffering! It was impossible to bring her here [to Belgium]! Maybe she would have been better cared for but I couldn’t tell the Ambassador: “my mother is sick”. Also, she didn’t have insurance here. She had insurance through her job [there] but it was worthless. We sent money but it’s our brothers who were in charge. They were careless. I took 3 weeks of my annual vacation last year to arrange the operation, find the best doctors [there] and make sure she was following the treatment. I sent money and call to make sure they did things right but it was too late (...).” (Solimar, 50, MDW, residing in Brussels).

Mobility strategies intersect with class status. The limited social capital of most Andean MDWs does not facilitate interactions with civil servants and social workers that could act as brokers to access social protection (Granovetter, 1983). In our fieldwork, informants who belong to ethnic minority groups in the homeland and worked for White employers abroad often experienced discrimination that limited their access to social protection in the receiving country. Lack of capital and adverse legal status can; however, be compensated under specific circumstances by a favorable relationship with the employer. Indeed, employers may also use their own social capital to the benefit of immigrants in order to help them or family members access healthcare. Marta –one of our informants— described such paternalistic practice in these terms:

“When my daughter was sick the Madame3 saw I wasn’t doing well. I didn’t know what to do or where to start up procedures to bring her. I don’t know who they got in touch with but 2 weeks later my daughter was here, getting treatment at the hospital [in Brussels] with the best specialist. She got cured” (Marta, 51, MDW, residing in Brussels)

Similarly, ethnic solidarity expressed through Catholic churches may compensate for the immigrants’ lack of access to formal care and weak family ties. Florencio for instance —a Colombian man who also held Spanish nationality—lacked health coverage in Belgium but church-goers organized a fund-raising to help him. Such practices are common amongst people who share the same hometown origin or belong to churches organized along ethnic lines.

A second way by which mobility is used to respond to healthcare needs within transnational families is when the immigrants’ themselves move temporarily or permanently to access formal healthcare coverage in the homeland. In this case, strategies of mobility and access to work-related health insurance are jointly used to respond to healthcare needs. Return migrants find that their return is facilitated by the possibility to benefit from another family member’s health insurance in the homeland. Immigrants and returnees however often perceive that the public health system in the homeland may not be of equal quality to that of their country of residence. In our fieldwork, this was particularly true for migrants of indigenous ethnic background who had experienced discrimination in the homeland’s health system. One of our informants named Valeria describes her ability to travel back to Colombia to receive care in these terms:

[My daughter] Lara’s work insurance [in Medellin] covers me. I mean like checkups and so on. But imagine I got cancer or something serious I would be forced to leave [Colombia again]. The co-pay is too high for me to pay [in Colombia]. [Also,] I would have to find out if my insurance covers dependents because I see Eduardo my husband is showing signs of Alzheimer (Valeria, 55, former MDW, residing in Medellin).

When mobility is impossible or perceived as too cumbersome to face a specific healthcare need, remittances are used as an alternative or a supplementary strategy to respond not only to the need of relatives but also to the long-term care needs of the immigrants themselves. As frequently noted in the literature, remittance may vary according to the gender of the sender and receivers (Levitt, 2001; Mazzucato, 2011). Because they fear that remittances sent to men will be diverted towards other priorities than the family’s well-being, Andean MDWs frequently select remittance-recipient in the homeland according to a gender and generational order. Trust is however not the sole criteria that guide aging migrant domestic workers to select younger female members of the family as remittance-recipient. Expectations about future care responsibilities towards aging migrants upon return are much more relevant. The relationship between Catalina —an aging MDW living in Belgium who suffers from serious hipbones issues— and her daughter-in-law Melinda who lives in Peru is telling in this respect:

Mrs. Catalina [her mother-in-law] is so nice! When baby Armandito is sick, she sends money to pay for the best doctors, because the [Peruvian] insurance we get through her son isn’t great. I know I would be there [for her] when she gets back. Helping her with the administration and so on. (Melinda, 24, daughter in law of MDW, residing in Lima)

After a brief visit in Peru to prepare her permanent repatriation in the homeland, Catalina felt relieved that her decision to trust Melinda with remittances would guarantee her access to care when she retires in Peru later on:

“ (…) [Melinda] is a really nice girl. When I got back she was there asking me: Mamita Catalina are you O.K? Do you need something? I don’t send the money to the boys directly because boys are boys at the end of the story we [women] take care of each other.”(Catalina, 50, MDW, residing in Brussels).

In this section, we have described the articulation between mobility, insurance, and remittances that form what we call sequential transnational social protection arrangements. Female MDWs who use these arrangements usually combine less privileged positions in racial and class terms. Their residence abroad and their family obligations in the homeland often add-up negatively and restrict their family’s and their own access to formal healthcare. This situation stimulates the use of remittances and community solidarity to access healthcare here and there. With sequential arrangements, it is primarily female members of the family who exchange items of equivalent value but at different points in time. As these families have restricted access to formal healthcare in sending and receiving societies, women have taken up the moral obligation to protect each other. It requires trust and bilateral balancing between particular actors here and there. Lastly, in sequential arrangements, social remittances play a key role as representations on the home and host country medical systems and past experiences with both systems push immigrants to opt preferably for European treatments whenever they are accessible.

### “Helping each other, sometimes”: Sporadic arrangements

Not all Andean MDWs in Belgium combine unfavorable ethnic and class positioning. In our fieldwork, we encountered numerous MDWs who belong to their homeland’s middle class and had achieved a high level of education prior to migrating. They left their country of origin during periods of economic and political hardship in search of professional opportunities. Those MDWs also tend to be regarded as White by other immigrants of the same national origin who belong to indigenous ethnic minorities. Their educational level, class, and ethnic positions provide them with a critical advantage when it comes to negotiating their family’s and their own access to health. This is visible at three levels. First, they deal more easily with the receiving country’s legal system which gives them not only greater chances of accessing a permanent legal status but also a better ability to exercise their rights to health. In addition to being less exposed to racial discrimination and better equipped to deal with bureaucratic challenges, they also benefit from personal networks of friends and relatives with a higher educational level that can act as a broker to access social protection. Natalia, for instance, is a Peruvian MDW who was a lawyer in her home country before she left for Spain 20 years ago. She left Spain following the 2008 economic crisis but had managed to secure citizenship before leaving. In Belgium, she is socializing primarily with highly-educated EU migrants. During an informal conversation, Natalia revealed how having grown up as upper-middle-class woman in Latin America helped her to access healthcare in Belgium:

“Charlie, a [Belgian] engineer I met in my French classes, told me all about the system here, how it works and how I could get my pneumonia taken care of; even as a Spanish citizen. He also took me to the best doctors. Thank God, I still kept some of my ability to move in the world and knew who to talk to”. (Natalia, 50, MDW, residing in Brussels).

Second, unlike immigrants involved in sequential arrangements, this second group of Andean MDWs can also extend more easily the benefit of their own social protection status to other family members in the homeland. Karla —who was looking for ways to grant her mother access to formal healthcare— gives an example of this privileged position within the underprivileged category of MDWs in Belgium:

My friends have always taken me to the right lawyers. I first brought [my mother] to Brussels with a (…)[temporary visa] and then I filed the procedure for a medical status residence. I met my friends through my ex-husband who was a political personality known in Colombia and abroad… They have always helped... (Karla, 51, MDW, residing in Brussels).

Third, because they belong to the middle and upper-middle class in the homeland, this second group of Andean MDWs can often count on a more substantial financial contribution of relatives in the homeland when designing their transnational health arrangements. This can even include the purchase of expensive insurances on the private market in view of future health needs in the family. The experience of Amelia, for instance, reveals how immigrants combine public and market-based resources upon designing their long-term transnational social protection strategies:

My mother has her pension in Lima; my father paid for it. When she was in Brussels, my husband and I paid for all her medical treatment. I mean she had access to insurance because I managed to regularize her [migratory] status when it was still possible. But here in Lima, Laureano my brother pays for her private health insurance, to have her cover when she returns. (Amelia, 45, daughter of former MDW, residing in Lima).

Because these migrants have more options in negotiating access to health for relatives and themselves, remittances are less frequent and do not play such a critical role to ensure reciprocal care as observed in the previous section. However, this second group of MDWs still uses remittances to respond to emergencies such as diseases, accidents or deaths of family members. Also, in spite of it’s favorable ethnic and class position, gender plays an equally strong role in the distribution of care responsibilities within this second group of MDWs. In other words, these families’ transnational social protection strategies still primarily work through direct interactions of female family members. Karla —a former teacher who left Colombia because of political persecution— described her infrequent use of remittances as follow:

I don’t help my other family members there [Colombia]. I guess this is because we came here for other reasons [than providing for our family]. My sister works and has a health insurance. We only send money for punctual emergencies. It’s mostly for the girls in Bogota. My niece had a car accident and we sent money to pay for her recovery treatment. But I mean these things we do more to be present since we can’t be physically there. (Karla, 51, MDW, residing in Brussels).

Because of their privileged access to social protection in destination countries and their lower use of remittances to address relatives’ health needs in the homeland, we qualify the healthcare negotiations of this second group as sporadic transnational healthcare arrangements. With sporadic arrangements, equivalence is less precise and the sequence of events is less narrowly bounded. In this case, the more favorable class, ethnic and educational positions of female migrants support a different system of reciprocity with relatives in the homeland. This particular standpoint also helps them counter-balance the effects of their underprivileged position as migrant domestic workers in Europe. Sporadic arrangements articulate favorable access to mobility for migrants and non-migrant relatives, recourse to market-based health solutions in the homeland and a more limited use of remittances. Within these arrangements, female migrants continue to play a critical role as organizers of the family’s access to health but —because of their more favorable class status in the homeland— support of other family members in the homeland (including men) is also more frequent. Unlike immigrants involved in sequential arrangements, this second group has fewer contacts with co-nationals and other Andean migrants through Church and tends to be active in migrant organizations that are not country-specific or in political organizations lobbying for immigrant rights in general.

## Conclusion: Combining intersectional and transnational approaches to study immigrant social protection

This article started by introducing a genuine typology of immigrant responses to their family’s and their own health care need in an era of increased transnational exchanges. In this sense, it has bridged the conceptual gap between rather static understandings of social protection as developed by social policy scholars and the unspecified strategies to access formal care discussed in previous work on transnational migration. Building on the concept of transnational social protection arrangement, we then discussed how immigrants articulate these different strategies according to different heterogeneity markers that operate in the various national contexts where transnational family networks are active. For our particular case study of Andean MDW, these markers were gendered responsibilities within the family, class, and ethnicity as experienced in the sending and receiving context but also religiosity, generational positioning within families and level of transnational engagement.

As we conclude this article, we maintain that combining the intersectional approach to social phenomena with the transnational approach to migration is a compelling conceptual tool to study immigrants’ inequalities in access to social protection. Indeed, existing studies have focused for too long on differentiated access to social protection within the boundary of a single nation-state basing themselves separately on heterogeneity markers that are gender, class or ethnic. Doing so, scholars have neglected that fact that—as noted by Faist et al. (2015)— new inequalities in access to social protection may be arising as immigrant families maintain cross-border connections. In this article, we have used an intersectional perspective to demonstrate how such transnational inequalities operate in the case of health. Examining multiple markers of difference, we noted that a privileged position in the homeland —such as belonging to the White middle class in Latin America prior to migration— can transform into an asset when it comes to ensuring immigrants and family members’ access to social protection here and there. As our data revealed, former professionals in Latin America now working as MDW in Europe are unsurprisingly in a better position to arrange access to social protection for themselves and for relatives than other MDW of the same national origin who have an indigenous ethnic background.

Yet, our data also showed that specific standpoints in one space may help compensate for socio-economic downward mobility experienced in another space. Indeed, Andean MDW with lower levels of education and belonging to ethnic minority groups were, in some circumstances, able to compensate for their disadvantaged position. Ethnic and religious organizations, as well as remittances, are critical resources in this respect as they serve as investments to ensure reciprocal care in case of necessity; therefore ensuring long-term access to social protection.

Overall, the case of Andean transnational families’ access to formal healthcare provided an illustration of the added value of our analytical approach. Beyond the study of access to healthcare in a context of South-North migration, combining transnational and intersectional approaches seem equally relevant for other types of migratory regimes (e.g. high-skilled migration and EU mobility) and for different areas of social protection than health (e.g. pensions and education). This contribution, therefore, contributed to consolidate transnational studies as an actual theory of society rather than just a mere description of networks that cross multiple nation states (Glick-Schiller, 2005, p. 439). At the same time, it also modestly contributed to increasing the explanatory power of intersectionality in a world of increasing human mobility (Mahler et al., 2015, Freznoza-Flot & Shinozaki, 2017, Anthias, 2008, Purkayastha, 2010). However, we are also firmly convinced that further research combining intersectional and transnational analytical lenses is needed to refine our understanding of immigrants’ access to social protection and, more generally, identify new types of inequalities that affect migrants and non-migrants alike.

## Abbreviation

MDW: Migrant domestic worker

## Footnotes

1 In Belgium, for instance, there are officially 21.084 Latin American registered. 60% of them are women. Colombians (around 5000) and Peruvians (around 2000) are among the most numerous groups and are also characterized by an over-representation of women (DGSIE, 2010), 2- MDW is the acronym for Migrant Domestic Worker; 3-The French word “Madame” used during the interview conducted in Spanish refers to her employer.
